# Effect of bFGF and fibroblasts combined with hyaluronic acid-based hydrogels on soft tissue augmentation: an experimental study in rats

**DOI:** 10.1186/s40902-019-0234-0

**Published:** 2019-11-06

**Authors:** Su Yeon Lee, Yongdoo Park, Soon Jung Hwang

**Affiliations:** 1 Department of Oral and Maxillofacial Surgery, Dental Research Institute, School of Dentistry, Seoul National University, 101, Daehak-ro, Jongno-gu, Seoul, 110-768 South Korea; 2 Department of Biomedical Engineering, Korea University Medical College, Seoul, Republic of Korea; 3 HSJ Dental Clinic for Oral and Maxillofacial Surgery, Wannam Building 2,3F 349 Gangnam-daero, Seocho-gu Seoul, 06626 Republic of Korea

**Keywords:** Hyaluronic acid, Hydrogel, Restylane, Fibroblast, bFGF, Soft tissue augmentation

## Abstract

**Background:**

Hyaluronic acid (HA) has been applied as a primary biomaterial for temporary soft tissue augmentation and as a carrier for cells and the delivery of growth factors to promote tissue regeneration. Although HA derivatives are the most versatile soft tissue fillers on the market, they are resorbed early, within 3 to 12 months. To overcome their short duration, they can be combined with cells or growth factors. The purpose of this study was to investigate the stimulating effects of human fibroblasts and basic fibroblast growth factors (bFGF) on collagen synthesis during soft tissue augmentation by HA hydrogels and to compare these with the effects of a commercial HA derivative (Restylane®).

**Methods:**

The hydrogel group included four conditions. The first condition consisted of hydrogel (H) alone as a negative control, and the other three conditions were bFGF-containing hydrogel (HB), human fibroblast-containing hydrogel (HF), and human fibroblast/bFGF-containing hydrogel (HBF). In the Restylane® group (HGF), the hydrogel was replaced with Restylane® (R, RB, RF, RBF). The gels were implanted subdermally into the back of each nude mouse at four separate sites. Twelve nude mice were used for the hydrogel (*n* = 6) and Restylane® groups (*n* = 6). The specimens were harvested 8 weeks after implantation and assessed histomorphometrically, and collagen synthesis was evaluated by RT-PCR.

**Results:**

The hydrogel group showed good biocompatibility with the surrounding tissues and stimulated the formation of a fibrous matrix. HBF and HF showed significantly higher soft tissue synthesis compared to H (*p* < 0.05), and human collagen type I was well expressed in HB, HF, and HBF; HBF showed the strongest expression. The Restylane® filler was surrounded by a fibrous capsule without any soft tissue infiltration from the neighboring tissue, and collagen synthesis within the Restylane® filler could not be observed, even though no inflammatory reactions were observed.

**Conclusion:**

This study revealed that HA-based hydrogel alone or hydrogel combined with fibroblasts and/or bFGF can be effectively used for soft tissue augmentation.

## Background

During recent decades, many techniques and materials for soft tissue augmentation have been introduced. Small deficiencies in the dermal or subdermal layer can be augmented by autogenous fat cells/tissue, allogenous dermal components or synthetic biomaterials to improve orofacial function and esthetics [1]. As the longevity of human life is lengthened and the importance of social interrelationships is increased, the interest in the improvement of orofacial function and esthetics has increased. Because of these interests, the need for less traumatic surgical techniques and more safe tissue substitutes have elevated drastically, which has led to the development of injectable fillers [2]. Numerous new fillers with different compositions have emerged in recent decades because of the ongoing requests for more efficient fillers [3, 4].

Biomaterials for soft tissue augmentation should be biocompatible, have a supportive function for soft tissue regeneration, especially collagen synthesis, and maintain an acceptable volume for as long as possible [5]. Ideally, it is desirable that biomaterials used for fillers be replaced by new soft tissue in the recipient site [6, 7]. Although many biomaterials used for soft tissue augmentation have been introduced and are commercially available, each of them has disadvantages, especially in the short-term. No fillers have shown satisfactory retention of volume or replacement by new soft tissue. Restylane® (Q-Med., Uppsala, Sweden) is a stabilized, partially cross-linked hyaluronic acid (HA) gel synthesized via the Streptococcus species biofermentation process [8]. Due to its biocompatibility, it is now one of the most frequently used fillers on the market. Although this form has been shown to be relatively safe and convenient to handle, its rapid resorption requires repeated injections [9].

Hydrogels consist of cross-linked polymers, and several natural or synthetic materials, such as gelatin, fibrin glue, HA, chitosan, silk fibroin, alginate, and polyethylene glycol, have been applied as hydrogels [10–17]. They have been used for scaffolds for engineered tissue repair or as carriers in drug delivery systems [15, 18, 19]. HA, a non-sulfated glycosaminoglycan (GAG), is one of the major components of the extracellular matrix and is found in all connective tissues of the body. It is a naturally derived, linear, high molecular weight polymer with visco-elastic properties [20]. Due to its excellent biocompatibility, HA is a preferred substance for hydrogel tissue regeneration and growth factor delivery [21].

Recently, HA-based hydrogels containing cultured fibroblasts and fibroblast-conditioned media have been applied experimentally [22–24] and clinically [25, 26], and they have shown improved efficiency [27]. It is known that basic fibroblast growth factors (bFGF) can induce the migration of mesenchymal stem cells and human fibroblasts [28, 29]. However, few studies have evaluated the effect of bFGF on soft tissue synthesis, especially collagen I synthesis. Moreover, the effect of bFGF combined with fibroblasts has not yet been reported. In this study, an HA-based hydrogel combined with fibroblasts and bFGF was used for soft tissue augmentation. The first aim of this study was to evaluate the effect of the HA-based hydrogel on soft tissue augmentation. The second aim was to investigate the effects of fibroblasts and bFGF added to HA-based hydrogels on the enhancement of soft tissue regeneration, which was compared with that of a commercial hyaluronan gel filler (HGF; Restylane®).

## Methods

### Materials

HA-based hydrogels were supplied from a biomedical engineering laboratory (Medical College of Korea University, Seoul, Republic of Korea). The brief manufacturing process [15] is described below. The commercial hyaluronan gel filler (HGF; Restylane®, Q-Med., Uppsala, Sweden) was donated from Contackorea Inc. (Seoul, Republic of Korea). Human fibroblasts were purchased from the Korean Cell Line Bank (KCLB, Seoul, Republic of Korea), and human bFGF was purchased from R & D Systems (Minneapolis, MN, USA).

### Preparation of the hydrogels

HA (0.25 mmol, based on the repeating unit MW) was dissolved in 40 ml of distilled water, and EDC (0.24 g, 1.25 mmol), 1-hydroxybenzotriazole hydrate (0.17 g, 1.25 mmol), and adipic acid dihydrazide (ADH) (2.2 g, 12.5 mmol) were added to the solution. The EDC-mediated coupling reaction between the carboxyl group of HA and the hydrazide group of ADH was conducted with stirring at room temperature for 8 h. HA–ADH was dialyzed against 100 mM NaCl for 2.5 days and distilled water for 1 day using a dialysis membrane (MWCO 14,000, SpectraPor; Rancho Dominguez, CA, USA). N-acryloxysuccinimide (NAS) (0.5 g, 3 mmol) was subsequently added to the HA–ADH solution. The reaction was conducted with stirring at room temperature for 12 h. HA–ADH–NAS was dialyzed extensively against 100 mM NaCl for 2.5 days and distilled water for 1 day. The product was then lyophilized for 3 days to obtain solid acrylated HA (HA-Ac). The degree of acrylation was calculated by comparing the peaks from the acryl and methyl groups in the HA residue. For the gel preparation, the acrylated HA was dissolved in a triethanolamine-buffered solution (0.3 M, pH 8). Polyethylene glycol tetra-thiols (MW 10,000) were added as cross-linkers with the same molar ratios of acryl and thiol groups. The HA-based hydrogel was formed via a Michael-type addition reaction. The reaction mixture was incubated at 37 °C to induce gelation.

### Culture of fibroblasts

To prepare the human fibroblasts, CCD-986sk cells (human fibroblast cells, KCLB, Seoul, Republic of Korea) were maintained in Dulbecco’s modified Eagle’s medium-F12 (DMEM-F12, Welgene Biotechservices Inc. Daegu, Republic of Korea) supplemented with 20% fetal bovine serum (FBS; Gibco, Grand Island, USA) and 0.5% gentamicin at 37 °C in 5% CO_2_.

### Preparation of the test conditions

The Hydrogel group was subdivided into four test condition groups: (1) hydrogel (100 μl) only (H) as a control, (2) hydrogel containing bFGF (10 μg/ml) (HB), (3) hydrogel containing human fibroblasts (1.0 × 10^5^ cells) (HF), and (4) hydrogel containing human fibroblasts and bFGF (HBF). For HF, 1.0 × 10^5^ human fibroblasts were suspended in Dulbecco’s phosphate-buffered saline and then dispersed in the hydrogel to form 100 μl of human fibroblast-mixed hydrogel, while bFGF (10 μg/ml) was also added for HBF.

The HGF group was also subdivided into four test condition groups similar to the hydrogel groups: (1) HGF (100 μl) only (R) as a control, (2) HGF containing bFGF (RB), (3) HGF containing human fibroblasts (RF), and (4) HGF containing human fibroblasts and bFGF (RBF). All preparation processes for each test condition were identical to those used for the hydrogel group.

### Animal experiments

All the animals were treated and handled in accordance with the ″Recommendations for Handling of Laboratory Animals for Biomedical Research″ compiled by the Committee on Safety and Ethical Handling Regulations for Laboratory Experiments at the School of Dentistry at Seoul National University. The animals were housed separately in temperature-controlled (22 °C) cages with a 12-h day/night cycle, and there were no restraints or food restrictions.

For this study, 10 athymic nude mice (SRC Inc., Japan) were used as host recipients for both groups (five for the hydrogel group and five for the HGF group). All animal experiments were performed after general anesthesia via the intraperitoneal injection of zolazepam (Zoletil, Virvac Lab., France, 30 mg/kg) mixed with xylazine (Rumpens, Bayer Korea Ltd., Republic of Korea, 10 mg/kg).

The HGF was commercial injectable filler; the four conditions used in the HGF group were induced by injection with a 1 ml plastic syringe with a 27 G needle. Because the manufacturing process used for the hydrogel used in this study did not take into account the need for injection, the viscosity of the gel obtained after adding the bFGF and/or fibroblasts was too thick for the 27 G needle used to inject the gel into the subdermal layer of the nude mice. Therefore, the hydrogel was placed into a 1 ml plastic syringe and cut into discs with 0.1 ml volumes after overnight gelation. Then, the discs were inserted into surgically generated subdermal pouches.

After the four test conditions were separately prepared, 0.1 ml of hydrogel corresponding to each condition was implanted into the subdermal layer on the back of the nude mice. The four conditions were injected or inserted randomly into four subdermal sites; two sites were on the left side of the vertebral line and two sites were on the right side. HGF (0.1 ml) was injected into the subdermal layer without any incisions after the subdermal insertion of the needle tip 1 cm from the vertebral line. For the hydrogel group, a small stab incision was made along the vertebral line, and a subcutaneous pouch was made by generating a 1-cm-long tunnel on the lateral side. Then, each of the four types of hydrogel was inserted into one of the four pouches, and the wound was sutured with 5–0 Surgisorb (Samyang Co., Republic of Korea).

The sites where hydrogel or HGF were placed were marked with a non-erasable pen (Namepen, Monami Co., Republic of Korea). Eight weeks later, the animals were sacrificed, and the four sites were excised wide enough to include skin beyond the boundary of the implanted materials.

### Histological analysis

The specimens were fixed in 10% buffered formalin, embedded in paraffin, sectioned into 4-μm sections, and stained with hematoxylin and eosin (H&E) and Masson’s trichrome (MT) stain. The digital images from the stained sections were obtained by means of a transmission and polarized light Axioskop Olympus BX51 microscope (Olympus Corporation, Tokyo, Japan). Tissue reactivity to the implanted materials was assessed by qualitative and quantitative histological evaluation. The qualitative analysis included the histological examination of tissue responses, the degradation of implanted material, the presence of capsules, and the tissue or cell infiltration of the surrounding tissue. The quantitative analysis was based on the measurement of the area of newly formed soft tissue within the hydrogel using the computerized image analysis system SPOT version 4.1 (Diagnostic Instrument, Inc., MI, USA) and ImageJ [30]. The percentage (%) of new soft tissue was determined as the ratio of the newly formed soft tissue area versus the total inserted hydrogel area. The ratios were statistically analyzed with Student’s *t* test.

### RT-PCR

Because we inserted human fibroblasts and bFGF into the nude mice, the gene expression of collagen type I in the implanted materials was examined using RT-PCR with human-specific primers. The results were normalized to the mRNA level of human GAPDH.

Total RNA was extracted by adding 0.5 ml of TRIzol® reagent (Invitrogen, Life Technologies, USA) to N_2_-frozen nude mice tissues. Each μg of RNA was subjected to cDNA synthesis by using SuperScript™ Reverse Transcriptase II (Invitrogen) and oligo (dT)12–18 primers (Invitrogen) in a 20 μl reaction volume according to the manufacturer’s instructions, with the additional step of removing the RNA complementary to the cDNA using *E. coli* RNase H (Invitrogen). One microliter of each cDNA was then subjected to polymerase chain reaction (PCR) according to the following amplification profile: predenaturation at 94 °C for 40 s, amplification (denaturation at 94 °C for 40 s; annealing at 60 °C for 40 s; extension at 72 °C for 1 min) for 30 cycles, and a final extension at 72 °C for 10 min in a DNA thermal cycler (model PTC-200, MJ Research, Inc., MA, USA). For each of the PCR products, 10 μl was electrophoresed on a 1.5% agarose gel in the presence of ethidium bromide and visualized by the Gel Documentation System (Vilber Lourmat, France).

## Results

**Fig. 1:**
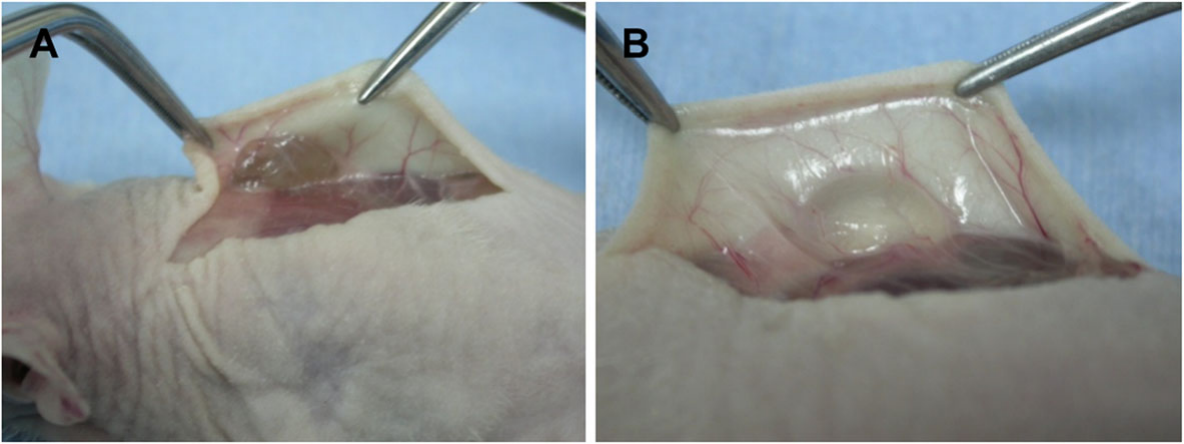
Macroscopic view. Hydrogel (**a**) and hyaluronan gel filler (**b**) groups just before sacrifice (8 weeks after surgery). The material remained as a yellow-white mass

Upon gross inspection, all the implanted materials in both groups remained well-defined nodules (Fig. 1). The HGF hydrogels were elastic and resembled rubber balls due to the presence of fibrous capsules, and the HA-based hydrogels were soft in texture.

### Histological evaluation

**Fig. 2:**
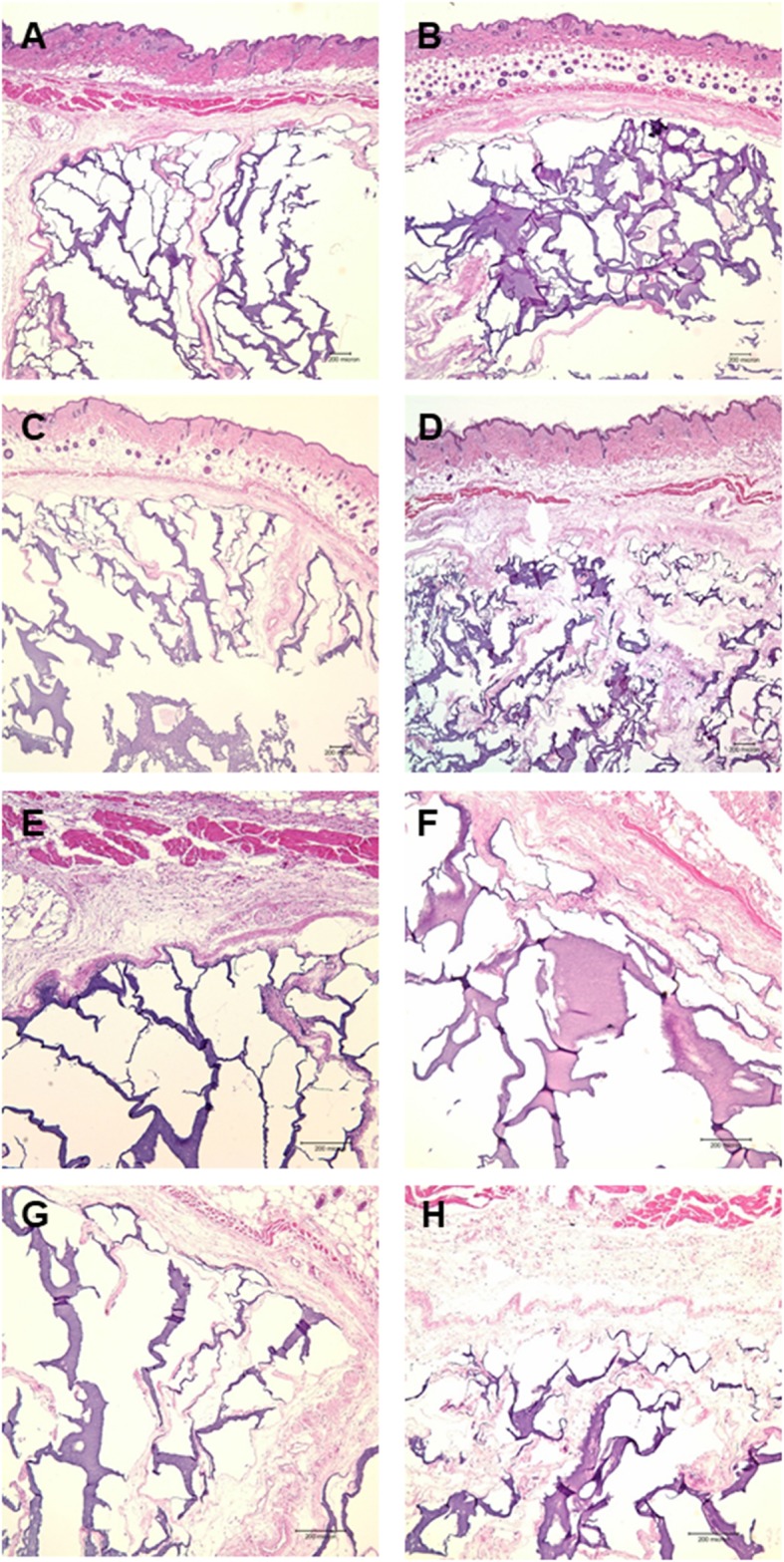
Microscopic view of the hydrogel groups stained with HE. **a**, **e** The hydrogel only group showed the active infiltration of cells and the ingrowth of host tissue into the hydrogel (HE; original magnification, × 40, × 100). **b**, **f** Hydrogel combined with bFGF produced more abundant fibrous soft tissue extending from the surrounding tissues into the hydrogel compared to hydrogel alone (HE; original magnification, × 40, × 100). **c**, **g** Hydrogel combined with fibroblasts also produced invaginated host tissue and collagen matrices (HE; original magnification, × 40, × 100). **d**, **h** Hydrogel mixed with bFGF and fibroblasts induced the most abundant fibrous matrix (HE; original magnification, × 40, × 100)

**Fig. 3:**
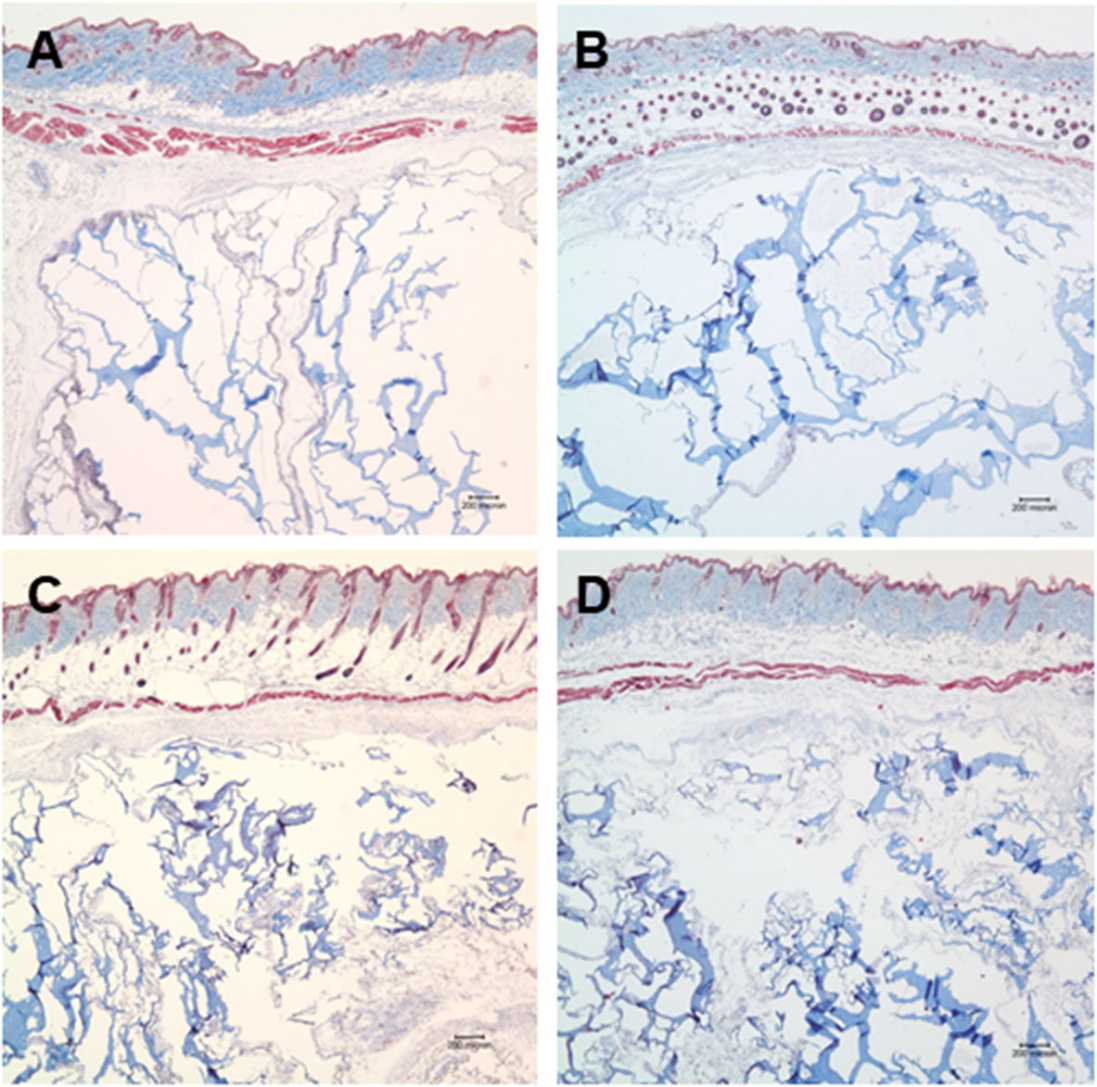
Microscopic view of the hydrogel groups stained with Masson’s trichrome stain. All soft tissues inside the hydrogel were stained blue (Masson’s trichrome; original magnification, × 40). **a** Hydrogel alone, **b** hydrogel combined with bFGF, **c** hydrogel combined with fibroblasts, and **d** hydrogel mixed with bFGF and fibroblasts

All hydrogels for each of the conditions were surrounded by a dense fibrous capsule. The infiltration of cells and host tissue ingrowth into the hydrogels and the collagen matrix inside the hydrogels were observed. Fibrous soft tissues from the surrounding tissues invaded the hydrogel, which broke the gel into pieces with an irregular pattern (Fig. 2). In the newly formed soft tissue, the collagen matrix was stained a deep violet by HE (Fig. 2) and blue by MT staining (Figs. 3).

**Fig. 4:**
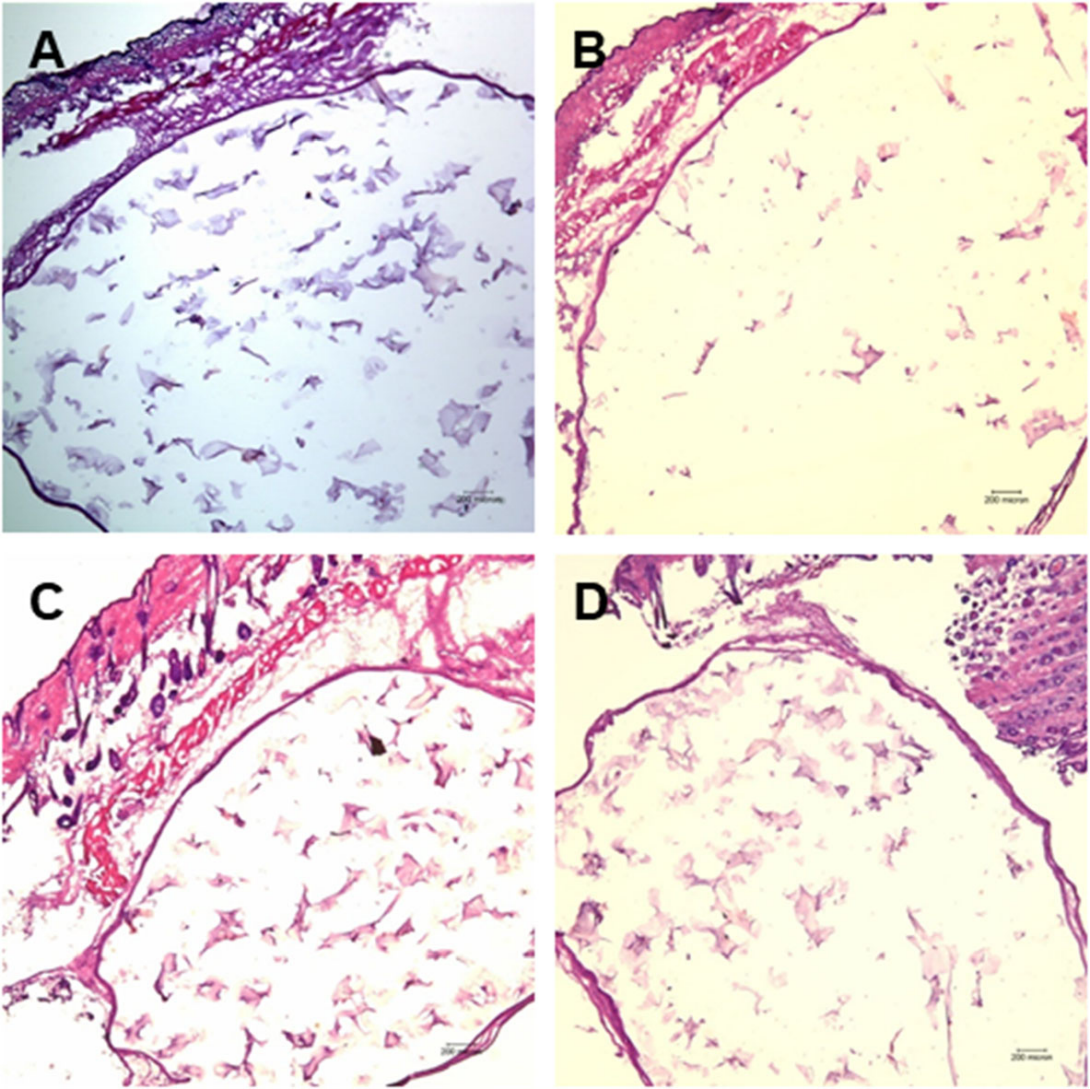
Microscopic view of the hyaluronan gel filler (HGF) groups stained with HE. All groups showed no soft tissue regeneration. HGF remained surrounded by a thin fibrous capsule (HE; original magnification, × 40). **a** Hyaluronan gel filler alone, **b** hyaluronan gel filler combined with bFGF, **c** hyaluronan gel filler combined with fibroblasts, and **d** hyaluronan gel filler mixed with bFGF and fibroblasts

Although the test condition of H did not involve bFGF or cells, soft tissue ingrowth was relatively more evident (Fig. 2, a and e), while HGF (R) did not present any new soft tissue formation (Fig. 4, a). In the test conditions with added bFGF or cells, soft tissue infiltration or synthesis was clearly increased, and it was the most prominent in the HBF condition, where the invaded soft tissue and formed collagen matrix were well intermingled in the hydrogel (Fig. 2).

**Fig. 5:**
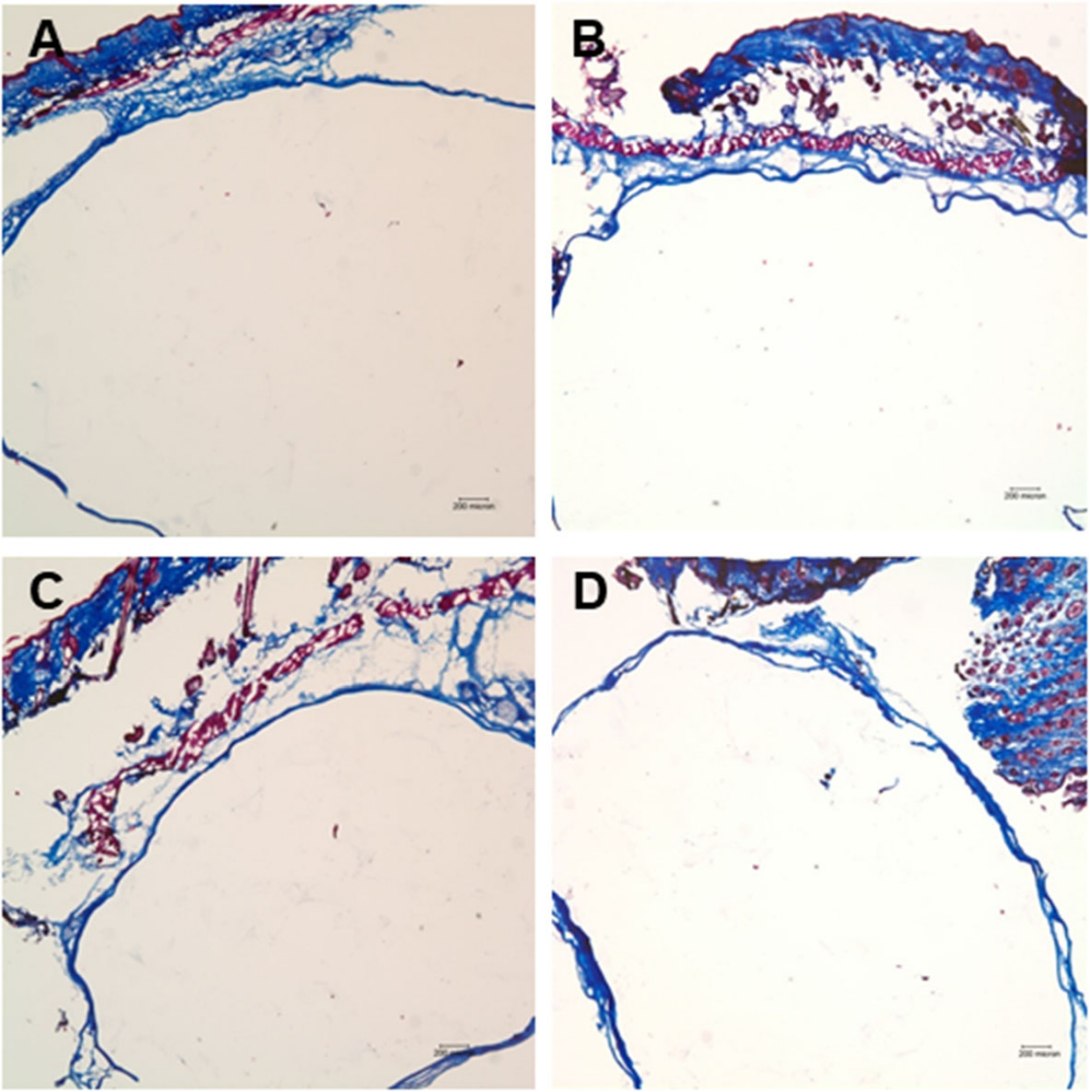
Microscopic view of the hyaluronan gel filler (HGF) groups stained with Masson’s trichrome stain. There was no soft tissue staining inside the filler material (Masson’s trichrome; original magnification, × 40). **a** Hyaluronan gel filler alone, **b** hyaluronan gel filler combined with bFGF, **c** hyaluronan gel filler combined with fibroblasts, and **d** hyaluronan gel filler mixed with bFGF and fibroblasts

In contrast to the soft tissue infiltration or synthesis observed in the hydrogel group, the implanted HGF failed to form new soft tissue. All implanted materials were surrounded by a uniform fibrous capsule consisting of three to four layers of fibroblasts with dense collagen fibers. Host cell infiltration and host tissue in-growth were almost absent in the R group (Figs. 4 and 5). Moreover, the human fibroblasts and/or bFGF added to the RB, RF, or RBF groups did not induce any proliferation or matrix synthesis (Figs. 4 and 5).

### Histomorphometric evaluation

**Fig. 6:**
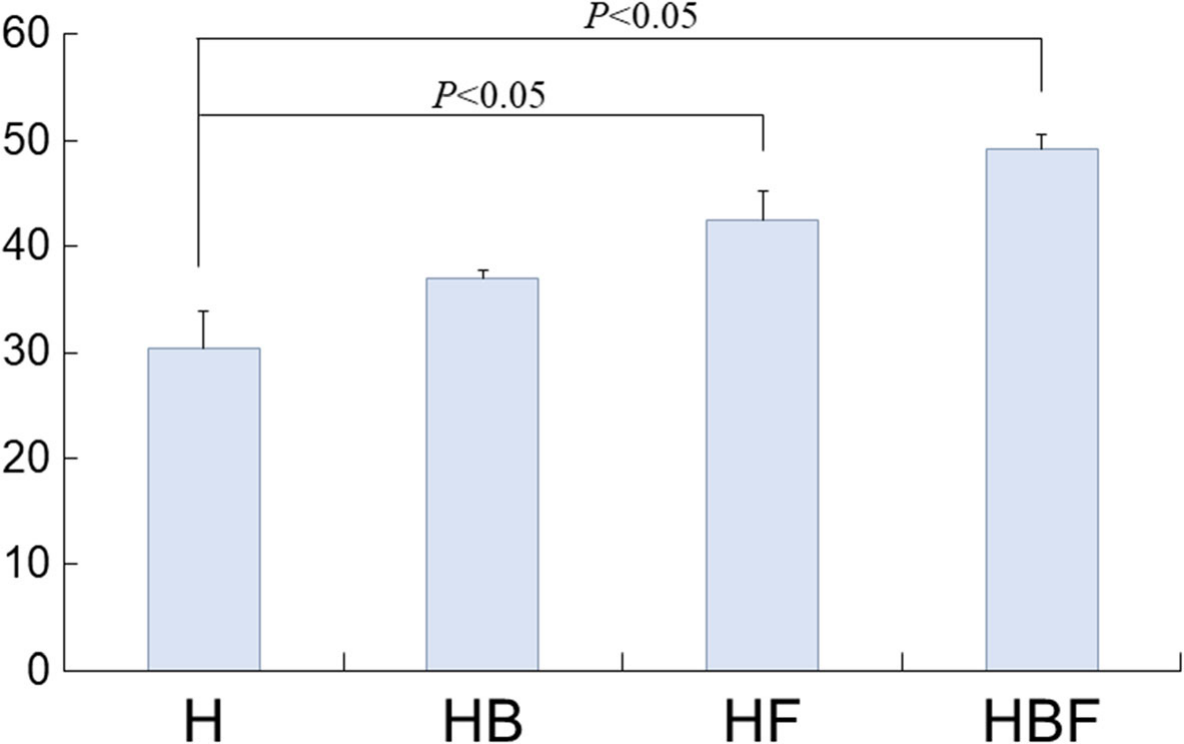
Soft tissue histomorphometry of the hydrogel groups. The amount of newly formed soft tissue was significantly higher in the hydrogel combined with fibroblasts (HF) and hydrogel mixed with bFGF and fibroblasts (HBF) groups compared with that in the hydrogel (H) group (*p* < 0.05)

Because there was no soft tissue ingrowth and no fibrous matrix formation inside the HGF hydrogels, the area of newly formed soft tissue could not be calculated in the HGF group. Therefore, it was analyzed only in the hydrogel group. The mean ratios of the newly formed soft tissue area within the hydrogels versus the total hydrogel area are presented as percentages (%) (Fig. 6). The ratios were increased in the following order: H (30.4%), HB (37.0%), HF (42.5%), and HBF (49.2%); HF and HBF showed significantly increased soft tissue formation compared to H (*p* < 0.05).

### Gene expression of collagen type I

**Fig. 7:**
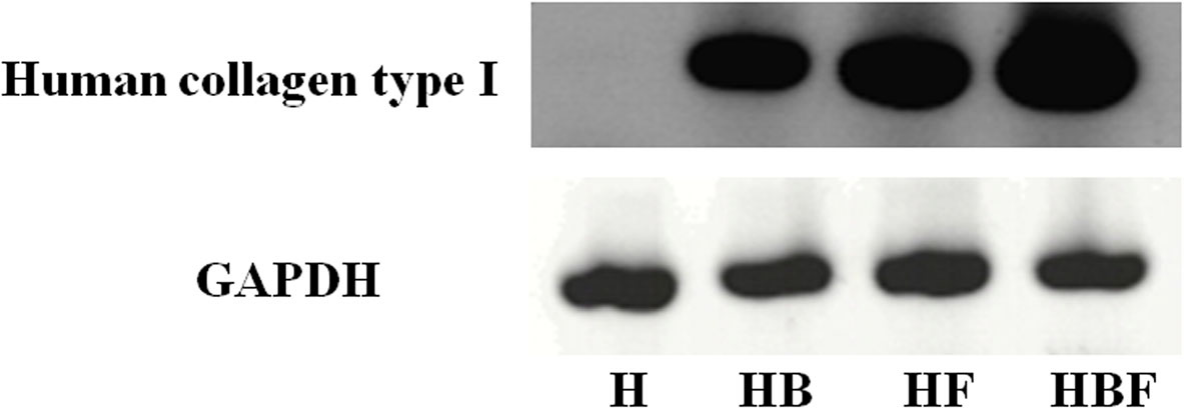
RT-PCR of human collagen type I. Human type I collagen was detected in hydrogel combined with bFGF (HB), hydrogel combined with fibroblasts (HF), and hydrogel mixed with bFGF and fibroblasts (HBF). GAPDH was used as an internal control. H: hydrogel only

Human collagen type I was well expressed in HB, HF, and HBF, and HBF showed the strongest expression. Although human fibroblasts were not present, HB unexpectedly expressed the human collagen type I gene (Fig. 7).

## Discussion

Many materials are now available for soft tissue augmentation. Autologous fat and dermal grafts have been preferred, but donor site morbidity and the high resorption rate (up to 70%) [31–33] limit their use. Xenogenic and allogenic collagen-derived dermal fillers, such as bovine collagen and human collagen, have been frequently applied in clinics and are regarded as the main commercially available fillers on the market [34]. However, their clinical use is decreasing due to the many problems arising from their non-autogenic properties, namely, the possibility of recipient hypersensitivity and the risk of infectious disease transfer [5, 35].

Recently, HA derivatives have been introduced for soft tissue augmentation. There are several commercial products on the market, such as AcHyal (Tedec Meiji Farma, Madrid, Spain), HydraFill (Allergan, Irvine, CA), Hylaform (Genzyme, Framingham, Mass), Captique (Genzyme and Allergan), Juve’derm (Allergan), Restylane (Q-Med, Uppsala, Sweden), Purogen (Mentor, Santa Barbara, CA), and Rofilan Gel (Rofil Medical International, Breda, Netherlands). Among the HA derivates, HGF (Restylane®) is the most popular because of its prior Food and Drug Administration approval, satisfactory safety margin, and recently reported the large consensus of approval [2]. There are many studies on HGF regarding its biocompatibility and adverse effects. Some authors have reported that HGF showed little foreign body reaction in human skin [36]. Other authors have reported that HGF underwent minimal cell infiltration in the surrounding fibrous capsules in a rat model and considered HGF to be biocompatible [6]. Similar results were shown in this study. The HGF implant remained surrounded by the fibrous capsule without any interactions with the host tissues. There was minimal cell infiltration into the implants.

HA-based hydrogels have been used in tissue engineering for drug delivery systems, wound healing, and tissue regeneration of nerves, bones, and cartilage [37, 38]. However, there have been few studies about the application of HA-based hydrogels to soft tissue augmentation. For other kinds of hydrogels, there have been efforts to apply hydrogels as biomaterials or scaffolds for soft tissue regeneration. A covalently linked, heparin-containing GAG hydrogel was used for the controlled release of growth factors during soft tissue regeneration in vivo and showed potential as a new biomaterial [39]. Agarose gel was evaluated in an animal model and considered a biocompatible product as a dermal filler [40]. Some hydrogel fillers are already available on the market. Polyacrylamide hydrogel has been approved for facial contouring in many countries under the brand name Aquamid (Contura S.A., Montreux, Switzerland) [41, 42]. Bio-Alkamid (Polymekon, Milan, Italy) has also been introduced as a polyalkylimide hydrogel filler [43, 44]. HA-based hydrogels were evaluated as soft tissue augmentation materials in this study. HA-based hydrogels showed high bioactivity. Hydrogel alone induced the ingrowth of fibrous tissues into the gel, demonstrating that hydrogel alone, without cells or growth factors, could be applied for soft tissue augmentation. These histological characteristics of HA-based hydrogels are similar to those of other kinds of hydrogels reported in previous studies. Maler et al. showed that there was a significant stromal invasion into gels with alginate that was gelled before injection and alginate with the cell adhesion tripeptide RGD (alginate-RGD) in a rat model [45]. Other authors demonstrated that alginate-RGD subcutaneous implants could support tissue and vascular ingrowth into gels in animal models [46, 47]. Fernandez et al. investigated the biocompatibility of agarose gel as a dermal filler and showed dense cellular infiltration and mature collagen bands inside agarose implants in a rat model [40].

It has been reported that growth factors and cells, especially fibroblasts and bFGF, function in tissue regeneration at various stages and play an important role in angiogenesis and wound repair [48–52]. Recently, there have been many efforts to apply fibroblasts and growth factors to soft tissue regeneration [39, 53–56]. There have been studies about the long-term corrective effects of injectable soft tissue fillers mixed with cells or growth factors [57, 58]. Autologous dermal fibroblasts mixed in HGF were used for nasal augmentation, and their clinical efficacy was evaluated in patients [57]. In another study, injectable alginate containing mesenchymal stem cells was studied in a rabbit model, and it was found to be a useful soft tissue augmentation material [58]. In this study, human fibroblasts and bFGF were mixed into hydrogel and HGF. The addition of fibroblasts improved the ability of the hydrogel to support the integration of surrounding tissue and the formation of the collagen matrix. The expression of human collagen type I was shown by RT-PCR in HB, HF, and HBF. It is known that bFGF can induce the migration of mesenchymal stem cells and human fibroblasts [28, 29]. Therefore, human collagen type I was expressed in HB in the present study, which suggests that bFGF could induce the migration of human fibroblasts from neighboring implanted material containing human fibroblasts (HF, HBF). HBF and HF showed a statistically significant increase in the percentage of soft tissue formation in the hydrogel. Similar results were noted by Marler et al., who demonstrated that the addition of syngeneic fibroblasts to alginate enhanced the gel construct volume in a rat model [45]. However, they concluded that this phenomenon seemed to be mediated by increased gel stiffness rather than by de novo tissue formation. In the present study, the addition of bFGF did not increase the amount of soft tissue in the hydrogel. Although there were no statistical significant differences between H and HB or HF and HBF, there was an increasing tendency of soft tissue formation in the hydrogel in the presence of bFGF. Yoon et al. reported that cultured human dermal fibroblasts mixed in HGF can survive and produce human dermal matrix [24]. This is not consistent with the results of the present study, in which there was almost no new soft tissue formation not only in R but also in RB, RF, and RBF; this may mean that human fibroblasts and bFGF did not have an active function within the HGF. This different result may be due to the use of different kinds of fibroblasts. A previous study used cultured fibroblasts from freshly minced healthy adult skin, while cultured fibroblasts from a cell line bank were used in this study.

## Conclusion

HA-based hydrogels are bioactive materials that integrate with the surrounding host tissues. With the addition of fibroblasts and/or bFGF, the hydrogels could produce human fibrous collagen matrices, and this was most apparent in hydrogels with fibroblasts and bFGF. Our findings suggest that HA-based hydrogel alone or in combination with cells and growth factors could be successfully used for soft tissue augmentation as a next-generation material.

## Data Availability

Not applicable.

## Abbreviations

ADH: Adipic acid dihydrazide
bFGF: Basic fibroblast growth factors
GAG: Glycosaminoglycan
H: Hydrogel only group as a control
HA: Hyaluronic acid
HB: Hydrogel added with bFGF
HBF: Hydrogel mixed with human fibroblast and bFGF
HE: Hematoxylin-eosin
HF: Hydrogel added with human fibroblast
HGF: Commercial hyaluronan gel filler, Restylane®
MT: Masson’s trichrome
NAS: N-acryloxisuccinimide
R: Restylane® as a control condition
RB: Restylane® added with bFGF
RBF: Restylane® mixed with human fibroblast and bFGF
RF: Restylane® added with human fibroblast
